# Supplementary material for: Quantifying the clusterness and trajectoriness of single-cell RNA-seq data
Source: PLoS Comput Biol. 2024 Feb 28;20(2):e1011866. doi: 10.1371/journal.pcbi.1011866 (PMC10927072; doi:10.1371/journal.pcbi.1011866)
Supplement: Attachment — Submitted filename: Response_to_reviewers_v14.docx [file pcbi.1011866.s001.docx]

**Response to the reviewers**
"Quantifying the clusterness and trajectoriness of single-cell RNA-seq data"
Hong Seo Lim, Peng Qiu

We would like to express our sincere gratitude to the reviewers of this manuscript. Your meticulous examination, insightful comments, and constructive feedback have played a pivotal role in enhancing the quality and rigor of this research. We greatly appreciate the time and effort you dedicated to reviewing our work. We have provided detailed responses to each of your suggestions, addressing concerns and incorporating valuable insights into the revision process.

**Reviewer #1**

Reviewer #1: The authors of this submission has presented to quantify "clusterness" and "trajectoriness" of scRNA-seq data, which could help researchers to decide what would be suitable analysis methods given scRNA-seq data. This review applauds the efforts of developing such a quantitative computational pipeline and believes that the presented work can indeed help researchers, especially biomedical researchers, to better explore and understand data.

There are a few questions/concerns that the authors may want to address:

1. Are the selected five metrics, pairwise distance distribution, persistent homology, vector magnitude, Ripley's K, and degrees of separation, comprehensive? Are there other possibly better metrics to use? The authors may want to discuss on the motivations of using these five specific metrics.

**We appreciate the reviewer's insightful comment regarding the selection of metrics in our study. The chosen metrics—pairwise distance distribution, persistent homology, vector magnitude, Ripley's K, and degrees of separation—were devised based on our intuition of their ability to effectively capture and differentiate geometric properties in cluster and trajectory data. Our motivation stemmed from the need to develop metrics that could discern relevant features in the context of scRNA-seq data. While we acknowledge that alternative metrics may exist, these five were tailored to address the nuances and differences that we felt could be effective in differentiating the two types of geometry here.**

**The key criterion for metric selection was their capacity to accurately classify the geometric properties of simulated and real scRNA-seq data. We aimed for metrics that not only provided meaningful insights into the underlying structures of the data but also demonstrated a decent level of discriminatory power. During our experimentation, these metrics consistently proved to be robust and effective in achieving accurate classifications, thus aligning with the overarching goals of our study.**

**Nevertheless, we appreciate the reviewer's suggestion and acknowledge that the field is dynamic, with continuous advancements in methodologies. In the revised manuscript, we have included a discussion on the motivations behind our choice of metrics, emphasizing the effectiveness of our selected metrics in achieving the desired classification accuracy for scRNA-seq data while acknowledging the possibility of alternative metrics. We believe this clarification will enhance the transparency and comprehensiveness of our methodology, addressing the reviewer's insightful input.**

As computing some of these metrics depends on some selected algorithms or hyperparameters, will the final "clusterness" and "trajectoriness" results change depending on these choices?

We would like to thank the reviewer for this comment. It is indeed useful to examine the robustness of our algorithm with respect to the hyperparameters. In this revision, we conducted a robustness analysis. We varied key hyperparameters associated with the scoring metrics. The hyperparameters we varied include:

- param1_num_bins_dpt (default=10, randomly chosen from [3, 5, 10, 15, 20])
- param2_num_bins_homology (default=2, randomly chosen from [2, 3, 5, 8, 10])
- param3_cells_per_cluster (default=20, randomly chosen from [10, 15, 20, 30, 40])
- param4_thresholdSizes (default=100, randomly chosen from [50, 80, 100, 120, 150])

We randomly generated 6 sets of parameter choices. For each of these 6 sets of parameter choices, we re-ran our entire analysis pipeline, re-computed the scoring metrics for the simulated datasets, re-generated the geometric landscape based on newly computed scores, and re-mapped the real scRNA-seq datasets to the simulated geometric landscape. The geometric landscape and the projections of real data are shown in below, for each of the 6 parameter settings. Compared to Figure 4 derived from the default parameter setting, results from these other parameter settings are similar. As shown in the figures below, the general structure of the UMAP landscape (projections of the simulated datasets) and the distribution of the red triangles (projections of real scRNA-seq datasets) look similar across different parameter choices.

We did not do many more of this mainly because of computational cost. Evaluating the 5 metrics for one dataset (size around ~5000 cells) takes about 45 seconds, which means that it takes about 150 hours to compute the scoring metrics for the 12,000 simulated datasets using one parameter setting. Although it is time consuming to compute the geometric landscape from the large collection of simulated datasets, we only need to it once. When this approach is applied to real data, the run time will be the time it takes to compute the metrics for this new data, which is about one minute.

Finally, it appears that higher degree of separation scores indicates better connectivity (or worse separation). Is this historically this way or it is better to call it degree of connectivity instead of separation?

**We agree with the reviewer's perspective that higher values of this score indicate better connectivity, and calling this score “degree of separation” can cause confusions. Indeed, the reason why we called it “degree of separation” is historical. Upon careful consideration, we acknowledge the merit of renaming this metric to better reflect its underlying concept. Moving forward, we will adopt the term "degree of connectivity" instead of "degree of separation"**

2. The generative 'geometric landscape' based on simulated data is using UMAP. Will the results depend on different embedding / projection methods

We appreciate the reviewer's inquiry regarding the potential dependence of our generative 'geometric landscape' on different embedding/projection methods. To address this concern, we applied the same analytical framework to two additional projection methods: tSNE and PCA. The simulated datasets underwent projection with each method, and subsequently, the scRNA-seq datasets were projected accordingly. The resulting geometric landscape and visualizations are shown below. From these visualizations, we can see that all three embedding approaches (UMAP, tSNE and PCA) are able to show good separation between the simulated cluster-like datasets and simulated trajectory-like datasets.

The projection of the real scRNA-seq datasets on the three embeddings show more differences. In the PCA embedding, the real scRNA-seq datasets do not project well onto the simulated geometric landscape, which is an indication that PCA is unable to fully capture the nonlinearity in the geometric landscape. To compare the differences between UMAP and tSNE, we examined the cluster/trajectory classifications of the real datasets, and generated the confusion matrices shown below. We observed a decent degree of consistency between UMAP and tSNE, with 119 out of 169 predictions being the same which constitutes 70 percent of the entire datasets.

Overall, our geometric landscape can be affected by the choice of embedding method. PCA seems insufficient, while UMAP and tSNE produce similar trend and predictions. The reason why we chose UMAP was motivated by its non-linear transformation capabilities, known preservation of global structure, computational efficiency, and reduced sensitivity to parameters. We believe the large collection and variation in simulated datasets represent a continuum of geometric properties, and UMAP’s ability to preserve global structure makes it a better choice to serve as the embedding method.

3. Such geometric landscapes can probably change with simulated data, for example, the smoothness of trajectories, the number of clusters, etc., which do not necessarily help derive consistent/useful "clusterness" and "trajectoriness" scores? How would the authors take care of these?

We agree with the reviewer's comment regarding the potential variability in geometric landscapes with respected to the simulated data. We have thought about this issue when we initially designed this project. To address this issue, we have considered diverse scenarios when we created the simulated datasets. For instance, when generating simulated cluster-like datasets, we used a range of values (2 to 7) for the number of clusters, as well as size/variance of the clusters and their constellations. When generating simulated trajectory-like datasets, variations in the smoothness of trajectories have been simulated by introducing varying levels of noise during the trajectory generation process. We believe that the diversity in the simulated datasets enables the generated geometric landscape to be consistent and representative, allowing us to derive useful "clusterness" and "trajectoriness" predictions for real datasets.

4. It is indeed encouraging to see the results in Figs 4 and 5. However, the authors may want to discuss some non-intuitive situations. For example, why some single clusters still appear in the right 'clusterness' branch in 'geometric landscape' in Fig 5a? In Fig 4b, it might be better to separately show the distributions of these metrics for "cluster"-like and "trajectory"-like scRNA-seq data to indeed check whether they are similar as simulated data, instead of pulling all 169 datasets together? Regarding the biological ground truth, are these labels in Fig 4c based on 20 PCs as described in the text or there are more biologically grounded knowledge?

We thank the reviewer for these detailed suggestions.

Regarding the question why some single clusters still appear in the “clusterness” region in Fig 5a: it's important to note that these single clusters are generated using Seurat's clustering pipeline with default parameter setting. It is possible that a Seurat-derived cluster may represent a cell type that contains distinct subpopulations, and the default resolution of Seurat did not separate the subpopulation into separate clusters. Therefore, this cluster itself can still be cluster-like, and appear in the “clusterness” region of the geometric landscape.

Regarding the comment of showing more details in Fig 4b, we have generated violin plots to separately show the distributions of the metrics for the four types of simulated datasets, the predicted cluster-like real datasets, and the predicted trajectory-like real datasets. The resulting figure is shown below. We can observe the concordance in trend between the simulated datasets and their counterparts in real scRNA-seq datasets. Although this figure below provides more details on the real scRNA-seq dataset, it provides overlapping information with Fig 2, and therefore, is partially redundant. After careful consideration of the flow of the paper, we decided to not include this figure in the revised manuscript.

Regarding the comment on biological ground truth labels in Fig 4c, these labels are obtained from the following publications:

- Saelens, W., Cannoodt, R., Todorov, H. & Saeys, Y. A comparison of single-cell trajectory inference methods. Nature Biotechnology 37, 547-554 (2019).
- Kim, T. et al. Impact of similarity metrics on single-cell RNA-seq data clustering. Briefings in Bioinformatics 20, 2316-2326 (2018).
- Krzak, M., Raykov, Y., Boukouvalas, A., Cutillo, L. & Angelini, C. Benchmark and Parameter Sensitivity Analysis of Single-Cell RNA Sequencing Clustering Methods. Frontiers in Genetics 10 (2019).

These references provided valuable biological insights that informed the ground truth labels. While we believe the determination of ground truths was guided by biological intuition, we acknowledge the possibility that some labels may also be influenced by computational analyses, as suggested by the reviewer.

5. In the generated landscape in Fig. 3, it is non-intuitive that both P-dist and degree of separation scores are high at the right-bottom tip. The authors may want to provide more discussions to help better understand the potential reason/limitation?

We appreciate the reviewer's observation regarding the generated landscape in Fig. 3. Upon closer examination, we agree that there is a slight increase of the “degree of separation”, or degree of connectivity As depicted in Fig 3a, the right-bottom tip region corresponds to simulated datasets that contain relatively larger multiple clusters. The increase of “degree of separation” metric in the right-bottom tip is because of the increased number of clusters.

**Reviewer #2**

Summary of Article

This article aims to address a key challenge in scRNA sequencing analyses. They discuss two key approaches to scRNA seq analysis: cluster analysis and trajectory inference analysis. Each of these approaches operate under distinct assumptions. This article aims to quantify the strength of each approach based on the geometric characteristics of the data. The authors present scoring metrics that aim to quantify “clusterness” and “trajectoriness” of the data. The authors use simulated data sets to differentiate between cluster-like data sets and trajectory-like datasets. They apply their metrics on real scRNA seq data sets to demonsrate how the metrics can be used as indicators of whether clustering or trajectory inference is appropriate for the data. This article would be of interest to the community of researchers using scRNA seq. It offers tools that can be used to help with the interpretation of data. With some revisions, this article is suitable for publication.

Methods:

The 5 scores that the authors use are well described in the methods and absolutely essential to understanding the manuscript though there are some inconsistencies between what the authors expect and the outcomes in the figures. Comments are below in the detailed notes.

**We would like to thank the reviewer for the concise summary and positive comment.**

The Github repository does share the code associated with the manuscript, but the README is empty and there is very little information on how to reuse the data set or run the analysis. Without a great deal of experience, this could would be difficult or impossible to reuse. The code would benefit substantially from additional annotation on what the different steps of the code are doing, order in which files would need to be used, etc. If someone had their own data set, where would it be brought into the pipeline so they can do their own comparison?

**We appreciate the reviewer's feedback on the GitHub repository, and we acknowledge the importance of making the code more accessible for potential users. To address this, we have incorporated two new Jupyter notebooks into the repository. The first notebook is designed to facilitate the reproduction of the results presented in the manuscript, including the generation of figures and key findings. The second notebook is specifically crafted to guide users in uploading their own datasets, enabling them to observe how their data would be projected onto the geometric landscape.**

**Furthermore, we recognize the necessity of providing clear instructions on replicating the necessary environments and installing the required packages. To enhance the user experience, we have supplemented the README file with detailed annotations, outlining the steps of the code, the order in which files should be utilized, and specific instructions for users with their own datasets. We believe these additions will significantly improve the usability of the code, allowing users, even those with limited experience, to effectively navigate and adapt it for their specific needs.**

Detailed Notes

Figure 1: Depiction of approach. No comments

Figure 2: Figure 2 depiction of score distributions grouped by set-type using simulated data sets. I agree this shows some indication of separation between clustered data sets and trajectory data sets.

Figure 3: UMAP clustering of 5-score outcomes from simulated data indicate separation between clearly clustered data sets and clearly trajectory data

3c – It appears that there is a gradient low-to-high for all metrics defined by the authors except for Homology and Degree of Separation. Can the authors comment on why these do or do not match the expected outcomes described in the methods section?

**Regarding the trend of the 5 metrics from cluster-like to trajectory-like, as shown in Figure 2, there is a low-to-high trend for 4 metrics except for Ripley’s K. In Figure 3c, we see that from right side (cluster-like) to left side (trajectory-like), 4 of the metrics showed blue-to-red color gradient, and Ripley’s K showed red-to-blue. Therefore, the trends in Figure 3c do agree with Figure 2 in general.**

**If we examine Figure 3c in more details, we can see additional patterns of variation of some metrics within one geometry type.**

- **For example, Degree of Separation metric is high on the left side (trajectory-like) and low on the right side (cluster-like) in general. If we focus on the right side, we see slight elevation of this metric in the bottom right tip, slightly higher than the rest of the right side. This is because the bottom right tip corresponds to cluster-like data with larger number of clusters.**
- **As another example, Homology metric is in general high on the left side and low on the right side. If we focus on the left side, we can see variation of this metric within the left side. This variation is correlated to the level of noise we simulated. Among the simulated trajectory-like datasets, larger amount of simulation noise (larger width of the trajectory) led to larger value of the Homology metric.**

**It is indeed an interesting discussion to explore how and why these metrics may vary within one geometry type (cluster-like or trajectory-like). We have included these descriptions in the result section.**

Figure 4: The authors depict where the real scRNA seq data sets fall along their UMAP spectrum of simulated data sets.

4a – Many of the data sets fall somewhere in the middle of the UMAP spectrum. It would be interesting to see a supplemental figure depicting some examples of scRNA seq data sets that fall in the middle where they are not exactly determined to be cluster like but also not exactly trajectory-like. In practice, someone might want to assess the clusteriness or trajectoriness characteristics in their data sets, but examples of how they might interpret data if it is not clearly one or the other would be very helpful for a reader.

4b – It appears as though the range of the data sets for all 5 metrics are similar between the simulated and scRNA-seq data sets, but the distributions are not all similar. The vector measurements and the degree of separation are particularly different. Can the authors comment on why the distributions are different between the scRNA seq and simulated data sets for these two metrics.

4c – The authors mention around a 70% agreement between the prediction of cluster-like/ trajectory-like geometry for real scRNA seq data sets and their presumed geometric intuitions based on the experimental design and underlying biology. It might benefit readers to see the scores and distributions of those that agreed versus the scores and distributions of those that did not agree. While the n is low for some of the presumed geometries, there could be enough data sets to display this for Cluster, Organs, Tree, Linear and possibly Bifurcation.

**4a - We appreciate the reviewer's suggestion to provide examples of scRNA-seq datasets that fall in the middle of the UMAP spectrum, where they are not definitively determined to be cluster-like or trajectory-like. On one hand, we agree that showcasing such examples is valuable for readers seeking to interpret data that exhibits characteristics of both cluster-like and trajectory-like patterns. On the other hand, the tools we can use to showcase such datasets are visualization tools such as UMAP, diffusion map and tSNE, coupled with cell clustering to add color. These visualization tools have their own assumptions and hence biases, and as a result, we see examples where clustering analysis produces clusters and trajectory inference produces trajectories. Therefore, the utility and interpretability of visualizing such borderline cases may be limited and difficult to justify. Nevertheless, we have included a jupyter notebook in our GitHub repo to show UMAP and diffusion map visualization of all the 169 benchmarking datasets, just in case readers are interested to see them.**

**4b - We agree with the reviewer’s observation that the range are similar between simulated and real data, but the distributions are not all similar. We believe this is reasonable. In our simulation, we defined 4 types (clear clusters, noisy clusters, clear trajectory, noise trajectory), and simulated equal number of datasets for each type. Our goal was to cover the universe of possible geometry, so that the subsequent UMAP geometry landscape can cover the possible geometry that we may see in any real scRNA-seq dataset. Therefore, the goal of the simulation was good coverage, not simulating the distribution of real data. The similar range is important, because it an indication that the simulated datasets achieved good coverage. Difference in distribution is okay because we did not intend to match the distributions between simulated and real data.**

**4c - The figure below was generated in response to a comment from reviewer one. In each panel, the first four violins shown the distributions of one metric in the four simulated types of dataset, showing how the metric varies between cluster-like and trajectory-like data. The last two violins show the distribution of the metric in real datasets, stratified by the predicted geometry. We can see consistent trend between simulated and real data. For example, simulated cluster-like data has lower P-dist compared to simulated trajectory-like data, real data predicted to be cluster-like also showed lower P-dist compared to real data predicted to be trajectory like. The same consistency can be observed for all five metrics. Although this is not exactly what the reviewer suggested, we feel this figure provides good insight that the scores/metrics are concordant with the predictions.**

Figure 5: Authors clustered the scores for the 169 scRNA seq data sets using Seurat. It is not entirely clear what this figure is adding to the manuscript. It appears the authors are adding an additional transformation to the data set but clustering in Seurat and seeing if it holds up in their geometric landscape. Since the data are already abstracted through the use of the scores, adding an additional layer of clustering feels difficult to interpret. I might rather see a comparison of score distributions for data sets that agreed compared to those that did not agree as mentioned in 4c.

**The purpose of Figure 5 is to provide further evidence that the proposed geometric landscape makes sense. We know that if we perform clustering on a cluster-like dataset, each of the resulting cluster should be less cluster-like compared to the original dataset. Similarly, if we perform clustering on a trajectory-like dataset, each of the resulting cluster should be less trajectory-like (e.g., trajectory becomes shorter) compared to the original dataset. In Figure 5, we see the dots (individual clusters) “moved” toward the upper-left noisy trajectory region compared to the triangles (original datasets) in Figure 4. This agreed with the expected effect of performing clustering. Therefore, this provided further evidence that the geometric landscape makes sense, and projecting real data to the geometric landscape is meaningful.**

**We understand why the reviewer felt this way. On one hand, we think it is reasonable to argue that Fig 5 does not add a huge amount of value to this article. On the other hand, the evidence shown in Fig 5 does not rely on experts’ manual intuition (which is needed to derive Fig 4c), or at least, Fig 5 rely on a different type of intuition. Therefore, Fig 5 provided a new argument to validate our approach, a new argument independent from Fig 4. That’s why we decided to include Fig 5 in this article.**

Figure 6: Representative examples of how real scRNA seq data sets described as trajectories and clusters fall into the geometric landscape. The examples are clear and illustrative.

Figure 7: Representative examples of how real scRNA seq data sets, that are not clearly described as trajectories or clusters, fall into the geometric landscape. The authors show that their method can help determine the clusterness and trajectoryness for data sets that are not clearly described as either.

Discussion: Overall, this manuscript is interesting. It offers tools to researchers to help with the determination of whether to use cluster analysis or trajectory inference analysis on a data set. While I agree with the authors that using one analysis will bias the outcome towards the type of analysis conducted (ie. clustering analysis will generate clusters) the authors could benefit from describing examples of where this could make a difference when studying the biological significance of different pathways. It does not feel sufficient to say that it will help us distinguish between tragectories of clusters or clusters of trajectories, why is this important biologically? It is always useful to have more tools to work with, but it remains unclear how this makes a difference in our fundamental study of biological systems.

**We appreciate the reviewer's summary of the Figures, as well as the discussions. Regarding the question of why this is important biologically, we believe there are several reasons: (1) If the data is cluster-like but is analyzed/interpreted by trajectory inference, one may derive an incorrect interpretation that distinct phenotype can transition into each other. (2) If the data is trajectory-like but is analyzed/interpreted as clusters, one may miss the fact that these resulting “clusters” are stages of a biological progression and can transition into each other. (3) When experimentally generating new scRNA-seq data, if one expects the data to be trajectory-like but the scoring metrics indicate the data is cluster-like (or vice versa), this could be an indication that something may have gone wrong in the experiment, or the initial assumption/intuition about the biology may be incorrect. In summary, quantification of clusterness and trajectoriness not only help us choose appropriate analysis and interpretation of scRNA-seq data, but also can serve QC purposes. We have included these points in the discussion section.**

**Reviewer #3:**

The authors provide a report that is centered around the development of a method/pipeline to evaluate / quantifiy “clusterness” and “trajectoriness” of scRNA-seq data. To achieve this, the authors propose five scores, namely pairwise distance distribution, persistent homology, vector magnitude, Ripley's K, and degrees of separation to quantify the “clusterness” and “trajectoriness” of single-cell RNA-seq data.

The subject is current and extremely relevant, and this beautiful study brings further understanding of the choices on how to analyze and interpret single-cell RNA-Seq data.

Since many of the single cell RNA-Seq datasets show both cluster-like and trajectory-like characteristics, I suggest that the authors apply clustering analysis to data showing this feature, and then evaluate the clusterness and trajectoriness for both the original dataset and the individual clusters. This would greatly improve the original manuscript.

**We appreciate the reviewer's suggestion. We would like to clarify that the results of the suggested analysis have been included in the manuscript, specifically discussed in the context of Figure 5.**

**We agree with the reviewer’s comment about the complexity inherent in scRNA-seq datasets, and the possibility that both cluster-like and trajectory-like characteristics may coexist. In fact, the concept of clusterness and trajectoriness, while informative, may not fully capture the rich geometry that scRNA-seq data can exhibit. For instance, datasets could manifest as "clusters of trajectories," where each distinct cluster itself represents a trajectory. Alternatively, a dataset might be a "trajectory of clusters," where spherical-shaped clusters are arranged in a constellation that forms a trajectory. These are some examples of possibilities where a dataset may exhibit both cluster-like and trajectory-like characteristics.**

**Aligned with the reviewer’s suggestion, we performed the analysis shown in Fig 5, where we performed clustering on real scRNA-seq datasets, treated each resulting cluster as an individual dataset, and projected the resulting clusters onto the geometric landscape. Our computational intuition is: (1) if we perform clustering on a cluster-like dataset, each of the resulting cluster should be less cluster-like compared to the original dataset; (2) if we perform clustering on a trajectory-like dataset, each of the resulting cluster should be less trajectory-like (e.g., trajectory becomes shorter) compared to the original dataset. The result of our analysis is shown in Figure 5. We observed that the dots (individual clusters) “moved” toward the upper-left noisy trajectory region compared to the triangles (original datasets) in Figure 4. This agreed with our intuition of the expected effect of performing clustering. Therefore, this provided further evidence that the geometric landscape makes sense, and projecting real data to the geometric landscape is meaningful.**
